# Increased Expression of miR-146a in Valvular Tissue From Patients With Aortic Valve Stenosis

**DOI:** 10.3389/fcvm.2019.00086

**Published:** 2019-06-26

**Authors:** Jana Petrkova, Jana Borucka, Martin Kalab, Petra Klevcova, Jaroslav Michalek, Milos Taborsky, Martin Petrek

**Affiliations:** ^1^Department of Pathological Physiology, Faculty of Medicine Dentistry, Palacky University, Olomouc, Czechia; ^2^Internal Medicine I - Cardiology, Palacky University and University Hospital, Olomouc, Czechia; ^3^Faculty of Medicine and Dentistry, Institute of Molecular and Translational Medicine, Palacky University, Olomouc, Czechia; ^4^Department of Cardiac Surgery, Palacky University and University Hospital, Olomouc, Czechia; ^5^Department of Clinical and Molecular Pathology, Faculty of Medicine and Dentistry, Palacky University, Olomouc, Czechia; ^6^Laboratory of Cardiogenomics, University Hospital Olomouc, Olomouc, Czechia

**Keywords:** aortic stenosis, microRNA, IRAK1, TLR4, epigenetics

## Abstract

miR-146a has been implicated in the regulation of the immune response as well as in inflammatory process of atherosclerosis. In the present study, we have investigated the expression of miR-146a and its targets, TLR4 a IRAK1, in aortic valve stenosis. A total of 58 patients with aortic stenosis (non- and atherosclerotic; tissue obtained during standard aortic valve replacement) were enrolled. The relative expression of mir-146a was higher in valvular tissue from patients with atherosclerosis compared to those without atherosclerosis (*p* = 0.01). Number of the IRAK1 and TLR4 transcripts did not differ between the investigated groups. There was a trend toward elevation of miR-146a expression in context of inflammatory infiltrate observed in the valvular tissue from patients with atherosclerosis (*p* = 0.06). In conclusion, in line with the acknowledged role of miR-146a in atherosclerotic inflammation, our data suggest it may be extended to the specific location of aortic valves in aortic stenosis.

## Introduction

Aortic valve stenosis represents the major cardiac valve disease which is characterized by inflammation, atherosclerosis, and calcification (1, 2). In addition to proatherogenic risk factors, mechanical forces, metabolic alteration, and environmental effects [rev. e.g., by Pasipoularides (3) and Cho et al. (4)], genetic and namely epigenetic mechanism have been recently nominated to play a role in aortic stenosis pathogenesis (5). In general, as reviewed by Menon and Lincoln (6) or Kishore and Petrek (7), epigenetic mechanisms exert their regulatory effects via processes of methylation, histone modification, and also activities of small non-coding RNAs—miRNAs.

Reflecting miRNAs effects as master regulators of gene expression in physiological and pathophysiological processes encompassing also cardiovascular system [reviewed e.g., by Kishore et al. (8)], it is relevant to address possible involvement of miRNAs as epigenetic factors involved in pathogenesis of aortic stenosis, including its atherosclerotic component. In this context, miR-146a could be a plausible candidate: apart from its proinflammatory/atherogenic properties (9, 10), miR-146a has been shown to be an important element in controlling signaling pathways including NF-kB, TRAF6, and IRAK1 (11, 12). These genes encode two key adapter molecules downstream of cytokine and Toll-like receptors (TLR) that have been involved in development of atherosclerosis (13), and recently also implicated in pathogenesis of aortic valve disease (14).

To investigate a plausible role of this candidate miRNA in aortic valve stenosis, we, therefore, investigated the expression of miR-146a in valvular tissues obtained from patients undergoing standard aortic valve replacement. We also determined the expression of Toll-like receptor (TLR)-4 and the interleukin-1 receptor associated kinase 1 (IRAK1) mRNA as plausible targets of miR-146a. Analyzing the obtained data, we wished to reveal if there was any relationship between miR-146a, their targets and pathological processes in valvular tissues.

## Materials and Methods

### Patients

Fifty-eight patients (for their characteristics see Table 1) with aortic valve stenosis have been enrolled, valvular tissue was obtained during standard aortic valve replacement (AVR); patients were enrolled on a consecutive basis, in time order of their AVR procedure. Presence of atherosclerosis was detected by angiography of coronary arteries prior the surgery; atherosclerosis was defined as more than 30% limitation of perfusion; 26 patients belonged to this category and 32 patients had non-altered perfusion. The patients who showed presence of inflammation, patients with systemic diseases and/or malignancies were excluded from the study. All patients have consented in writing to the participation in this study according to the Declaration of Helsinki and the Ethics committee of University Hospital and Faculty of Medicine, Palacky University Olomouc approved the study protocol.

**Table 1:** Characteristics of study groups.

**Parameter**	**Non-atherosclerotic**	**Atherosclerotic**
Number (no.) of patients	32	26
Age (years)	68.6 (7.6)	71.0 (8.1)
Sex (Males/Females)	18/14	20/6
Cholesterol (mmol/L)	4.78 (1.20)	4.51 (1.03)
Triglycerides (mmol/L)	1.56 (1.15)	1.46 (0.71)
HDL (mmol/L)	1.33 (0.42)	1.33 (0.29)
LDL (mmol/L)	2.74 (1.15)	2.53 (0.88)
Height (cm)	168 (9.5)	166.7 (10.4)
Weight (kg)	83.1 (12.8)	84.7 (15.6)
BMI	29.4 (5.0)	30.5 (5.8)
Smoking (absolute no./from all patients) (%)	5/32 (15.6)	5/26 (19.2)
Inflammation, absolute no./from all assessed specimen (%)	19/29 (65.5)	13/23 (56.5)
Fibrosis, absolute no./from all assessed specimen (%)	16/31 (51.6)	11/26 (42.3)

*Patients with aortic stenosis were divided based on assessment of coronary perfusion into two groups: those with non-altered perfusion (non-atherosclerotic) and with decreased perfusion (atherosclerotic), for details see section materials and methods*.

*If not specified otherwise, the values represent the mean and standard deviation (in brackets)*.

*Terms “Inflammation” and “Fibrosis” refers to valvular tissue*.

### miRNA/mRNA Determination

The valvular tissue obtained during surgery was placed into RNA later solution to prevent RNA degradation. Subsequently, total RNA was extracted from aortic valvular tissues by *mir*Vana™ miRNA Isolation Kit; whole (complete, homogenized) tissue specimen were used for the extraction procedure For the miRNA detection, RNA was reverse transcribed using TaqMan MicroRNA Reverse Transcription kit and TaqMan MicroRNA Assays using a specific reverse primer and real time PCR was performed with primer-probe with specific primers for miR-146a (all so far mentioned reagents/kits/primers were from Life Technologies, Thermo Fisher Scientific, Waltham, MA, USA) and LightCycler 480 Probes Master (Roche Life Sciences, Indianapolis, IN, USA). Expression levels of miR-146a were normalized to RNU6B (Life Technologies, as above). Real time PCR was performed in Rotor Gene detection system (Corbett Research, Mortlake, NSW, Australia). Program was set up for holding at for 10 min, followed by 40 cycles consisting of for 15 s and for 60 s. TLR-4 and IRAK1 mRNA expression was determined by the same methodology, the probes used for the assessment of this gene were (TLR4 left primer: CTCTCCTGCGTGAGACCAG, TLR4 right primer: CAGCTCCATGCATTGATAAGTAA; IRAK1 left primer: tgcctggtgtacggcttc, IRAK1 right primer: ctgaggccaggagagaggt) obtained from Roche Applied Science (Penzberg, Germany). Expression levels of TLR4 and IRAK1 were normalized to GAPDH (left primer: agccacatcgctcagacac, right primer: gcccaatacgaccaaatcc).

### Histopathological Examination

Tissue sections were evaluated by a histopathologist to assess presence of inflammatory infiltrate and fibrosis (absolute and relative values are shown in Table 1, bottom lines); the extent of infiltrate, if present, was semi-quantitated (grading + to ++++). Standard procedure utilizing hematoxylin-eosin staining in formalin-fixed paraffin-embedded (FFPE) samples was utilized.

### Statistical Analysis

The non-parametric Mann–Whitney *U-*test was performed to assess the relative expression of miR-146a, TLR4, and IRAK-1 transcripts and to test for differences between patient subgroups. Pearson's correlation coefficient was used to examine the relationship between miR-146a and TLR4 and miR-146a and IRAK1 expressions. *P* < 0.05 was considered statistically significant.

## Results

**Figure 1:**
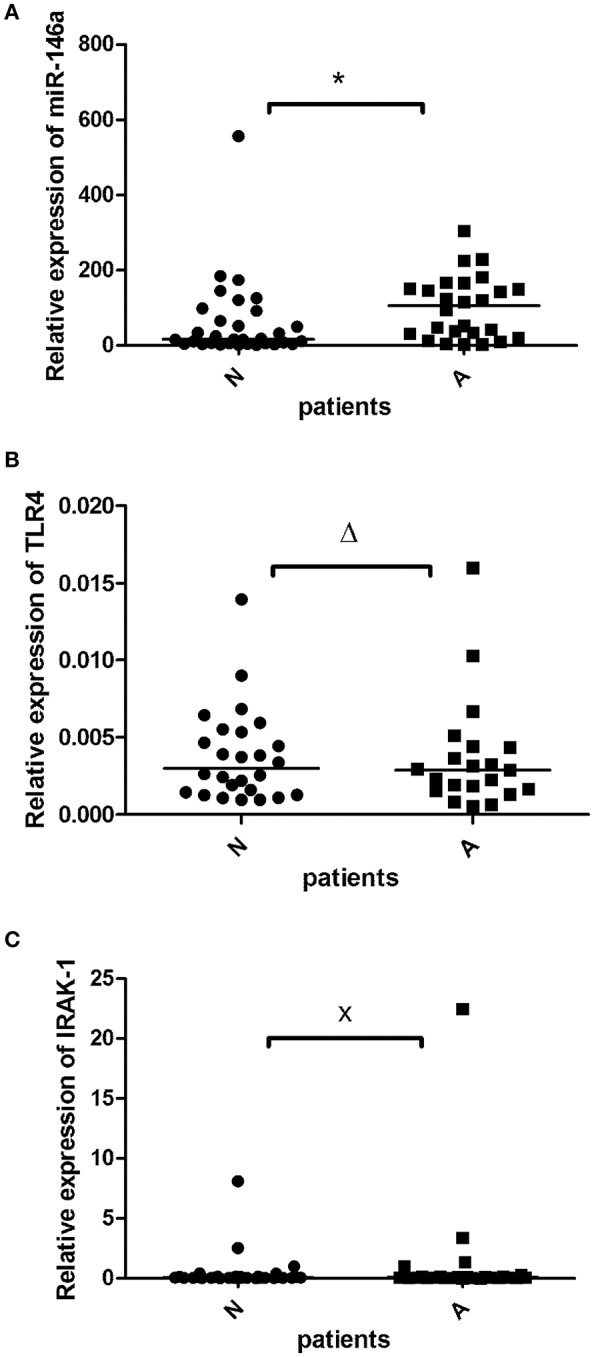
The relative expression of miR-146a **(A)**, TLR4 m-RNA **(B)**, and IRAK1 m-RNA **(C)** in the valvular tissue obtained from patients with aortic stenosis with signs of atherosclerosis (A; *n* = 26^≠^) and without atherosclerosis (N; *n* = 32^≠^). The miR-146a expression was normalized to the RNU6B expression, expression of TLR-4 and IRAK1 was normalized to GADPH. The lines represent median values and the following symbols denote *P*-values: * = 0.01; Δ = 0.64; x = 0.57. ^≠^Note: for TLR4 **(B)** *N* = 26 and *A* = 21 patients.

miR-146a was detected in 58 of 58 samples (100%). When the study subjects were separated based on angiography perfusion data into two subgroups, miR-146a was elevated in the aortic valve tissues from 26 patients with decreased coronary perfusion as a marker of atherosclerosis compared to 32 patients with non-altered perfusion, *p* = 0.01 (Figure 1A).

TLR-4 transcripts were detected in 47 of 58 samples (81%). TLR-4 mRNA expression did not differ between the atherosclerotic and non-atherosclerotic subjects (*p* > 0.05), Figure 1B. There was a trend to negative correlation (*r* = −0.2) between the miR-146a and TLR-4 mRNA expression (*p* = 0.1).

IRAK1 mRNA was detected in all 58 investigated samples (100%). There was no difference between IRAK1 mRNA relative expression in atherosclerotic and non-atherosclerotic subjects (*p* > 0.05), Figure 1C. There was no relationship between the miR-146a and IRAK1 mRNA expressions.

**Figure 2:**
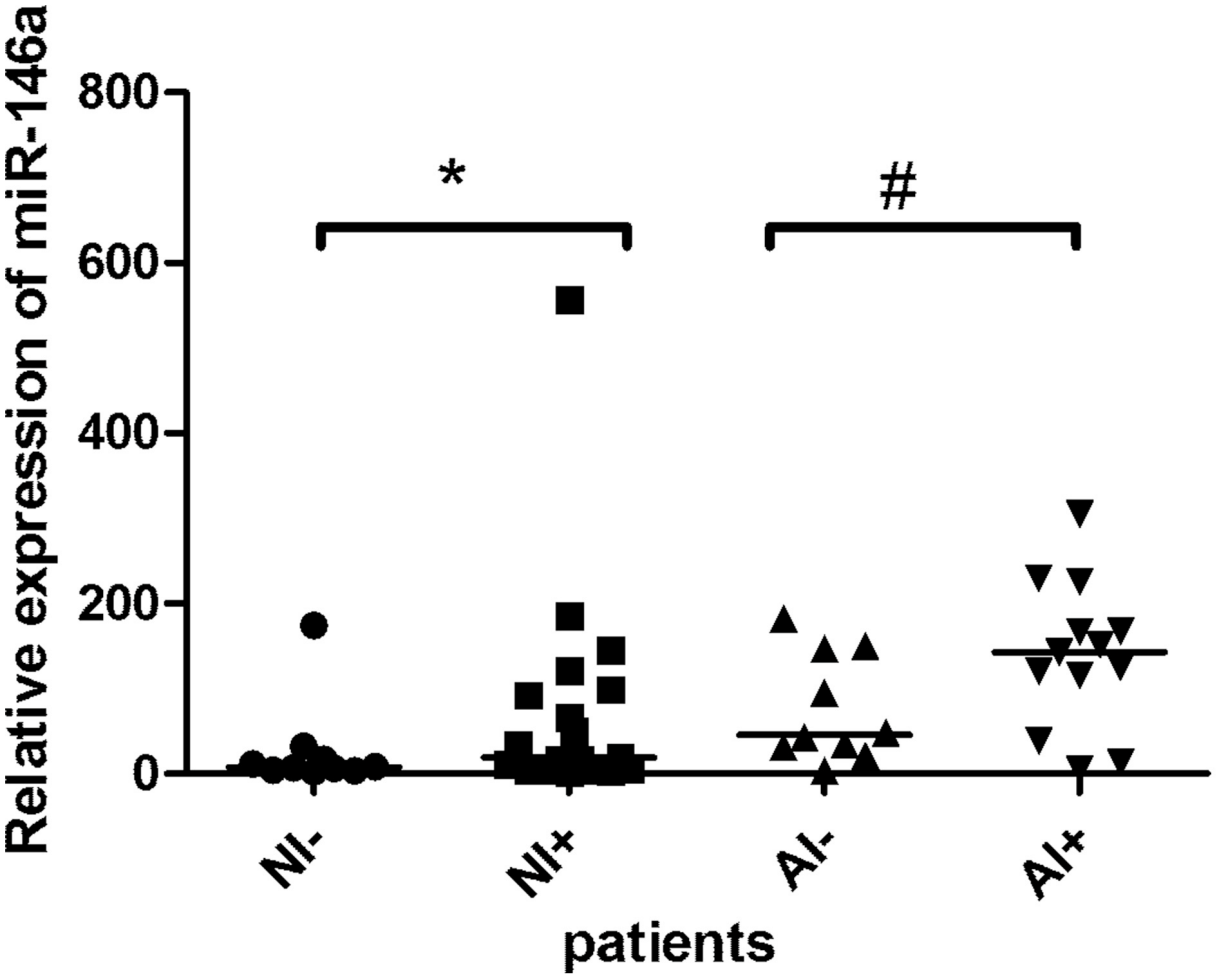
The relative expression of miR-146a in the valvular tissue obtained from patients with aortic stenosis. Description of abbreviations designating patient subgroups: non-atherosclerotic patients with (NI+, *n* = 19) or without inflammatory cell infiltrate (NI-, *n* = 10); atherosclerotic patients with (AI+, *n* = 13) or without inflammatory cell infiltrate (AI-, *n* = 10). The lines represent median values and the symbols denote *P*-values: * = 0.10, # = 0.06.

**Figure 3:**
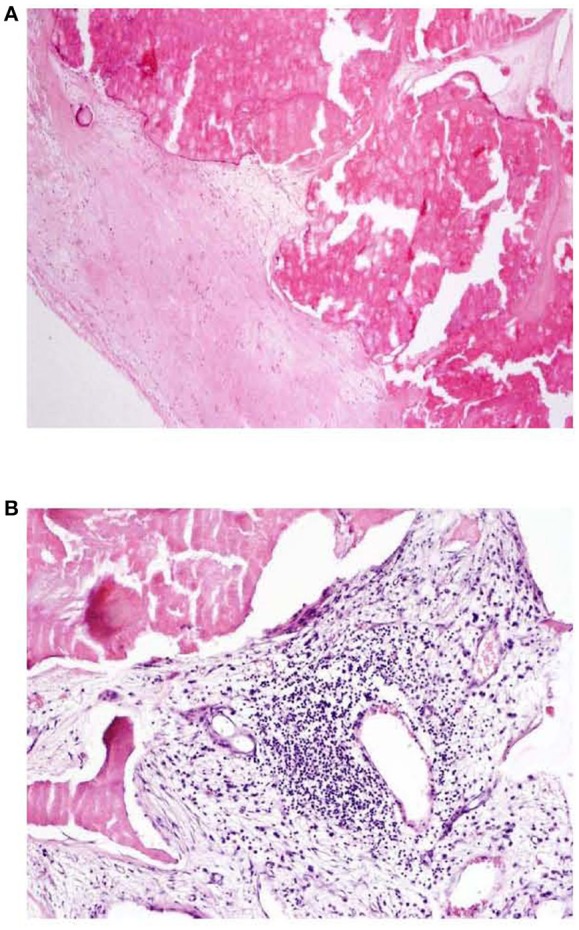
**(A, B)** Aortic valve tissue histopathology HE, 40x **(A)**, 100x **(B)**; **A**: dystrophic calcification of valve, absence of inflammatory infiltration, **B**: presence of mononuclear (lymphoplasmacytic) inflammatory infiltration in connective tissue of aortic valve.

When study subjects were further sub-grouped according to absence/presence of inflammatory cellular infiltrate, a trend toward miR-146a elevation was observed in patients with infiltrated valvular tissue (Figure 2), this observation was more pronounced in atherosclerotic patients (*p* = 0.06) than in patients without atherosclerosis (*p* = 0.1). Figure 3 shows representative examples of valvular histopathology.

## Discussion

This study investigated the expression of miR-146a and of its targets, TLR4 and IRAK1, in valvular tissue obtained from the patients with aortic valve stenosis. Upregulation of miR-146a expression in valvular tissue was observed in the subgroup of patients with decreased coronary perfusion as a marker of atherosclerosis. miR-146a expression tended to be elevated in those atherosclerotic patients whose valvular tissue contained inflammatory cell infiltrate. These findings extend the previous and recent reports (9, 10, 15, 16) about the role of miR-146a in atherosclerosis in general to the specific location in aortic valve stenosis. Overall, though obtained in a smaller scale study limited to patients, our data may implicate this non-coding miRNA, and in wider sense epigenetic factors, as eligible research targets in further investigations of pathogenesis of aortic valve disease.

miR-146a has been implicated in several key physiological processes including innate immune and inflammatory responses. It has been previously shown that upregulation of miR-146 family (miR-146a/b) regulated downstream toll-like receptor 4 (TLR4) signaling, IL-1 receptor associated kinase 1 (IRAK1), and TNF-receptor associated factor-6 (TRAF6) through a negative-feedback regulation loop (11, 17). In this context, we analyzed expression of target genes miR-146a, specifically of the *TLR4* and *IRAK1* genes. There was, however, no difference between expression of the TLR4 and also of the IRAK transcripts between the groups of atherosclerotic and non-atherosclerotic patients, only an insignificant inverse relationship between miR-146a and TLR4 miRNA expression could be described. While this observation is in contrast with some reports, e.g., of a correlation between miR-146a and TLR4, IRAK1 in patients with coronary artery disease (18), others have observed reduced expression of IRAK1 by upregulation of miR-146a, e.g., in psoriasis (19) and in senescent cells (20). It is, therefore, conceivable that expression and mutual relationship of miR-146a and its targets may reflect a specific localization of inflammatory processes. This suggestion implied from our primary analyses of miR-146a expression and TLR4 and IRAK transcripts should be, therefore, verified by further experiments, preferably with expanded collection of aortic valve samples, eventually of different stages, and also on protein level.

It should be also mentioned that the expression of both miR-146a and its targets is, on an individual basis, affected by variations in their gene sequences. Functional single nucleotide polymorphisms (SNPs) have been reported in *TLR4, IRAK1* genes (21, 22) and importantly they are also located in pre-miR146a—those were responsible not only for the establishment of diversity among individuals but also for changes in their expression and/or development of different disease phenotypes, including coronary heart disease (23–25).

Despite limitations of the present study (analysis of a single miRNA in a cohort comprising only patients with aortic valve stenosis, not subjects without this pathology), to our knowledge we present the first report plausibly implicating miR-146a in aortic valve stenosis. A spectrum of miRNAs, but not including miR-146a, has been recently found to be deregulated in patients with aortic stenosis [reviewed by Menon and Lincoln (6)]; miR-146a has also not been noted in microarray expression study combined with bioinformatics analyses (26), nor in a recent report by Duan et al. (27). In this context, it is desirable to conduct studies of miRNA-146a expression in extended cohorts including specimen from patients without valvular disease. These studies should address in greater detail presence and plausible role of miRNA-146a targets, preferably including *in situ* hybridization and/or immunohistochemistry experiments, also for characterizing the cellular infiltrate. If our data are validated and extended, there may be yet another plausible candidate for further studies of miRNAs involvement in aortal valve stenosis, which could also be explored on the level of exosomes and extracellular vesicles as recently proposed by Blaser and Aikawa (28).

## Ethics Statement

All patients have consented in writing to the participation in this study in accordance with the Declaration of Helsinki and the Ethics committee of University Hospital and Faculty of Medicine, Palacky University Olomouc approved the study protocol.

## Author Contributions

The idea to investigate miRNA expression in aortic valves was initiated by JP, who set up the clinical design and also provided the patients with the help of MK and MT. JP also realized the logistics within the laboratory and pathology department. JB performed the RT-PCR expression analyses and, together with JP and MP, drafted the manuscript. PK determined TLR4. JM performed histopathological examination. The final version of the paper was prepared by JP and MP. MP is the author responsible for the integrity of the data. All authors approved the final version of the manuscript.

### Conflict of Interest Statement

The authors declare that the research was conducted in the absence of any commercial or financial relationships that could be construed as a potential conflict of interest.
